# Stem Cells from Human Exfoliated Deciduous Tooth Exhibit Stromal-Derived Inducing Activity and Lead to Generation of Neural Crest Cells from Human Embryonic Stem Cells

**DOI:** 10.22074/cellj.2015.510

**Published:** 2015-04-08

**Authors:** Khadijeh Karbalaie, Somayyeh Tanhaei, Farzaneh Rabiei, Abbas Kiani-Esfahani, Najmeh Sadat Masoudi, Mohammad Hossein Nasr-Esfahani, Hossein Baharvand

**Affiliations:** 1 Department of Cellular Biotechnology at Cell Science Research Center, Royan Institute for Biotechnology, ACECR, Isfahan, Iran; 2 Department of Molecular Biotechnology at Cell Science Research Center, Royan Institute for Biotechnology, ACECR, Isfahan, Iran; 3 Department of Genetics at Reproductive Biomedicine Research Center, Royan Institute for Reproductive Biomedicine, ACECR, Tehran, Iran; 4 Department of Stem Cells and Developmental Biology at Cell Science Research Center, Royan Institute for Stem Cell Biology and Technology, ACECR, Tehran, Iran; 5 Department of Developmental Biology, University of Science and Culture, ACECR, Tehran, Iran

**Keywords:** Differentiation, Human Embryonic Stem Cells, Neural Crest Cells, SHED, SDIA

## Abstract

**Objective:**

The neural crest is a transient structure of early vertebrate embryos that generates neural crest cells (NCCs). These cells can migrate throughout the body and produce a diverse array of mature tissue types. Due to the ethical and technical problems surrounding the isolation of these early human embryo cells, researchers have focused on in vitro studies to produce NCCs and increase their knowledge of neural crest development.

**Materials and Methods:**

In this experimental study, we cultured human embryonic stem cells (hESCs) on stromal stem cells from human exfoliated deciduous teeth (SHED) for a two-week period. We used different approaches to characterize these differentiated cells as neural precursor cells (NPCs) and NCCs.

**Results:**

In the first co-culture week, hESCs appeared as crater-like structures with marginal rosettes. NPCs derived from these structures expressed the early neural crest marker p75 in addition to numerous other genes associated with neural crest induction such as *SNAIL, SLUG, PTX3* and *SOX9*. Flow cytometry analysis showed 70% of the cells were AP2/P75 positive. Moreover, the cells were able to self-renew, sustain multipotent differentiation potential, and readily form neurospheres in suspension culture.

**Conclusion:**

SHED, as an adult stem cell with a neural crest origin, has stromal-derived inducing activity (SDIA) and can be used as an NCC inducer from hESCs. These cells provide an invaluable resource to study neural crest differentiation in both normal and disordered human neural crest development.

## Introduction

Tooth pulp is comprised of living connective tissue that contains multipotent stromal cells which are commonly regarded as mesenchymal stem cells that have originated from neural crest cells (NCCs) (1,2). Dental pulp mesenchymal stem cells with stromal characteristics are a population of highly proliferative, clonogenic, multipotent cells capable of differentiation into a variety of cell types, including neural and non-neural cells (1). Stromal stem cells from human exfoliated deciduous teeth (SHED) present cranial neural crest and neural markers. These stromal cells innately express several neural markers and the expression of these markers increases upon neural induction (2). 

It is generally accepted that ectomesodermal lineage or NCC differentiation in human embryonic stem cells (hESCs) can be induced by co-culture with murine PA6 stromal cells (3,7). This observation has been further verified on another stromal cell line-MS5. This characteristic was named "stromal derived inducing activity (SDIA)" (8). Therefore, based on the origin of SHED cells from NCCs, we have hypothesized that SHED cells have SDIA in induction of NCCs from hESCs. 

## Materials and Methods

### hESC culture

We used the hESC line, Royan H5 (9), for this experimental study. The cells were passaged and maintained under feeder-free culture conditions in hESC medium that contained Dulbecco’s Modified Eagle Medium: Nutrient Mixture F-12 (DMEM/ F12; Invitrogen, USA) supplemented with 20% knockout serum replacement (KOSR, Invitrogen) and basic fibroblast growth factor (bFGF; 100 ng/ ml, Royan Institute, Iran). Cells were grown in 5% CO_2_at 95% humidity and passaged every seven days. hESCs were passaged by incubation in collagenase IV (1 mg/ml, Invitrogen), then mechanically fragmented into small pieces and replated on Matrigel (Sigma-Aldrich, USA). 

## SHED culture and co-culture methods

In this study, we collected children’s normal human exfoliated deciduous incisors following approval by the Institutional Review Board and Institutional Ethical Committee of Royan Institute. These SHED cells were isolated as previously described (2). SHEDs were plated in DMEM supplemented with embryonic stem cell-qualified fetal calf serum (ES-FCS; 10%, Invitrogen) on 6 cm collagen (Sigma-Aldrich)coated dishes at a concentration of 400,000 cells per dish for three days until confluency. Co-culture induction was initiated by plating approximately 400 hESC pieces on SHEDcoated dishes in Glasgow minimum essential medium (GMEM, Invitrogen) supplemented with 10% KOSR, nonessential amino acids (0.1 mM, Invitrogen), L-glutamine (2 mM, Invitrogen), sodium pyruvate (1 mM, Sigma-Aldrich, P4562) and β-mercaptoethanol (0.1 mM, Sigma-Aldrich). The culture medium was changed every other day after day 4. hESCs were allowed to differentiate in the co-culture system for 14 days. 

## Neural precursor cell (NPC) culture and proliferation

In order to obtain a NPC culture we used TrypLE™ Express (Invitrogen) to digest the day-14 co-cultures. Of note, these cells easily detached from the feeder layer. The detached cells were seeded at a density of 10000 cells/cm^2^in polyL-ornithine (0.2 mg/ml, Sigma-Aldrich, P4970) and laminin (20 µg/ml, Sigma-Aldrich) -coated dishes on neural precursor medium that consisted of DMEM/F12 supplemented with KOSR (5%), N2 supplement (2%, Invitrogen), bFGF (20 ng/ml), epidermal growth factor (EGF; 20 ng/ml, Sigma-Aldrich) and heparin (2 µg/ml, Sigma-Aldrich). The neurosphere culture was carried out at the same cell density and culture media on non-adherent tissue culture dishes (Greiner, Germany) with medium refreshment every third day. For additional passaging of the NPCs, we disaggregated the cells with TrypLE™ Express at day 2 for the adherent culture and day 7 for the non-adherent culture. Disaggregated cells were subsequently seeded on new dishes. 

## Karyotype analysis

For karyotype analysis, we treated the cells with 0.66 mM thymidine (Sigma-Aldrich) for 16 hours. After washing, the cells were left for 5 hours and then treated with colcemid (0.15 m g/ml, Invitrogen) for 30 minutes. Isolated cells were exposed to 0.075 M KCl (Merck, Germany) at 37˚C for 16 minutes and subsequently fixed in three consecutive immersions of ice cold 3:1 methanol: glacial acetic acid (Merk, Germany), then dropped onto pre-cleaned chilled slides. Chromosomes were visualized using standard G-band staining. At least 20 metaphase spreads were screened of which 10 were evaluated for chromosomal re-arrangements. 

## Neural differentiation

Neural differentiation was achieved by withdrawal of bFGF and EGF from the neural precursor culture medium. 

## Osteogenic differentiation

For osteogenic differentiation, precursor cells were seeded at 10000 cells per 35 mm culture dish and cultured for 3 weeks in the presence of 10 mM β-glycerol phosphate, 0.1 µM dexamethasone and 200 µM ascorbic acid (Sigma-Aldrich) inα-minimal essential medium (α-MEM; Invitrogen) medium that contained 10% FBS. 

The medium was refreshed every third day. The cultures were fixed using 4% paraformaldehyde (Sigma-Aldrich) and stained with alizarin red. 

## Immunocytofluorescence staining

Slides were fixed in 4% paraformaldehyde. For intracellular markers, permeabilization was carried out with Triton X100 (0.2%, Merck) and cells were incubated with primary antibodies diluted in PBS with bovine serum albumin (BSA; 5 mg/ml, Sigma-Aldrich). The appropriate fluorescein isothiocyanate (FITC), tetramethylrhodamine isothiocyanate (TRITC) and phycoerythrin (PE) -labeled secondary antibodies diluted with BSA (2.5 mg/ml, Sigma-Aldrich) and diamidino-2-phenylindole (DAPI; 3 ng/ml, Sigma-Aldrich) were used for cell visualization with a fluorescent microscope (Olympus, BX51, Japan). Primary and secondary antibodies are listed in table 1. 

## Reverse transcription-polymerase chain reaction analysis (RT-PCR)

Differentiated hESC colonies were mechanically excised from the SHED cell layer sheet by a syringe needle. These clamps were gently washed with PBS and the RNeasy Mini Kit (Qiagen, Spain) was used for extraction of total RNA. Prior to RT-PCR, RNA samples were digested with DNase I (Fermentas, Germany) to remove any contaminant genomic DNA. Standard RT-PCR was performed using 2 μg total RNA, oligo (dT) and the RevertAidTM H Minus First Strand cDNA Synthesis Kit (Fermentas) according to the manufacturer’s instructions. The cDNA samples were subjected to PCR amplification using human specific primers. Amplification conditions were as follows: initial denaturation at 94˚C for 5 minutes, denaturation at 94˚C for 30 seconds, annealing at 55-70˚C for 45 seconds. extension for 45 seconds at 72˚C (35 cycles) and a final polymerization at 72˚C for 10 minutes. PCRs were performed in triplicate. PCR products were electrophoresed in 1.7% agarose gels that contained ethidium bromide (10 μg/ml, Sigma-Aldrich). Bands were visualized by using a transilluminator (SynGene, Korea).

Real-time RT-PCR was performed using a Thermal Cycler Rotor Gene 6000 (Corbett, Australia) with 10 μl of SYBR Green PCR Master Mix (Takara, Germany), 0.25 μM of each primer, and 50 ng of cDNA for each reaction in a final volume of 20 μl. Cycle conditions were carried out according to the protocol by (Takara), relative gene expression was analyzed using the comparative cycle threshold (Ct) method, 2^-ΔΔCt^ (10). All samples were normalized to levels of GAPDH, which was used as the housekeeping gene. All measurements were performed in triplicate. Real-time specific primer pairs were designed by Beacon Designer software (Version 7.2, BD, USA). The sequence of primers and PCR conditions are presented in table 2.

Quantitative RT-PCR (qRT-PCR) data were presented as mean ± standard deviation (SD). 

**Table 1:** Antibodies used in this study


Primary antibody	Host	Company	Cat. #	Dilution	Antibody target

**ZO1**	Mouse	Milipore	339100	1:100	Epithelial tight junction molecule
**NESTIN**	Rabbit	Sigma	N5413	1:200	Intermediate filament protein
**PAX6**	Rabbit	Santacruz	SC-11357	1:200	Transcription factor
**OTX2**	Rabbit	Sigma	HPA000633	1:100	Transcription factor
**SOX1**	Rabbit	Sigma	S8318	1:50	Transcription factor
**RC2**	Mouse	Milipore	MAB5740	1:300	Radial glial cell marker
**Ki67**	Mouse	Milipore	MAB4190	1:200	Nuclear protein
**TH**	Mouse	Sigma	T1299	1:200	Cytoplasmic enzyme protein
**PERIPHERIN**	Mouse	Sigma	P5117	1:750	Type III intermediate filament protein
**TUJ**	Mouse	Sigma	T3952	1:200	Microtubule element
**NCAM**	Rabbit	Milipore	AB5032	1:200	Neural cell adhesion molecule
**BRN3A**	Rabbit	Sigma	B9684	1:200	Transcription factor
**AP2**	Mouse	Sigma	A7107	1:500	Transcription factor
**P75**	Rabbit	Sigma	HPA004765	1:600	Nerve growth factor receptor
**VIMENTIN**	Mouse	Abcam	Ab8978	1:20	Intermediate filamen type TIII
**IgG isotype control**	Mouse	Milipore	CBL600	1:200	Negative control
**IgG isotype control**	Rabbit	Abcam	Ab37415	1:1000	Negative control
**Secondary antibody**					
**FITC anti-rabbit IgG**	Goat	Sigma	F1262	1:60	
**FITC anti-mouse IgG**	Goat	Milipore	AP124F	1:50	
**TRITC anti-mouse IgG**	Goat	Sigma	T7782	1:50	


#; Number.

**Table 2:** Primer sequences and reverse transcription-polymerase chain reaction analysis (RT-PCR) conditions


Gene	Primer sequence (5΄→3΄)	AT (˚C)	Length (bp)	Cycle	Accession No.

**PAX7 **	F: AAGATTCTTTGCCGCTACCA R: CACAGTGCTTCGGTCACAGT	62	192	35	NM_002584.2
**PTX3 **	F: GTGGGTGGAGAGGAGAACAA R: TTCCTCCCTCAGGAAACAATG	60	175	35	NM_002852.3
**PAX6 **	F: CAGCTCGGTGGTGTCTTTG R: AGTCGCTACTCTCGGTTTA	60	214	35	NM_001127612.1
**SOX9 **	F: AGTGGGTAATGCGCTTGGATAGGT R: CGAAGATGGCCGAGATGATCCTAA	60	204	35	NM_000346.3
**MSX1/2 **	F: CCTTCCCTTTAACCCTCACAC R: CCGATTTCTCTGCGCTTTTCT	59	285	35	NM_001195262.1
**SNAIL2/ SLUG **	F: AGCGAACTGGACACACATAC R: TCTAGACTGGGCATCGCAG	58	411	35	NM_003068.3
**NESTIN **	F: TCCAGGAACGGAAAATCAAG R: TTCTCTTGTCCCGCAGACTT	55	564	35	NM_006617.1
**FOXA2 **	F: CCACCACCAACCCCACAAAATG R: TGCAACACCGTCTCCCCAAAGT	60	294	35	NM_021784.4
**SOX17 **	F: CGGTATATTACTGCAACTAT R: GGATTTCCTTAGCTCCTCCA	60	105	35	NM_022454.3
**BRACHYURY**	F: AATCCTCATCCTCAGTTTGG R: GTCAGAATAGGTTGGAGAATTG	60	141	35	NM_003181.2
**GATA4 **	F: CCTGTCATCTCACTACGG R: GCTGTTCCAAGAGTCCTG	60	181	35	NM_002052.3
**FOXD3 **	F: CAAGCCCAAGAACAGCCTAGTGAA R: TGACGAAGCAGTCGTTGAGTGAGA	66	203	35	NM_012183.2
**AP2 **	F: TCCCTGTCCAAGTCCAACAGCAAT R: AAATTCGGTTTCGCACACGTACCC	50	396	35	NM_001122948.1
**SOX1 **	F: CCTCCGTCCATCCTCTG R: AAAGCATCAAACAACCTCAAG	60	201	35	NM_005986.2
**c-KIT **	F: GCGAGAGCTGGAACGTGGAC R: CTGGATGGATGGATGGTGGTGGAGAC	62	174	35	NM_000222.2
**c-RET **	F: CCGCTGTCCTCTTCTCCTTCATC R: GCTTGTGGGCAAACTTGTGGTAG	62	81	35	NM_020630.4


AT; Annealing temperature.

## Results

### SHED cells induce neural fate in hESCs

**Fig.1:**
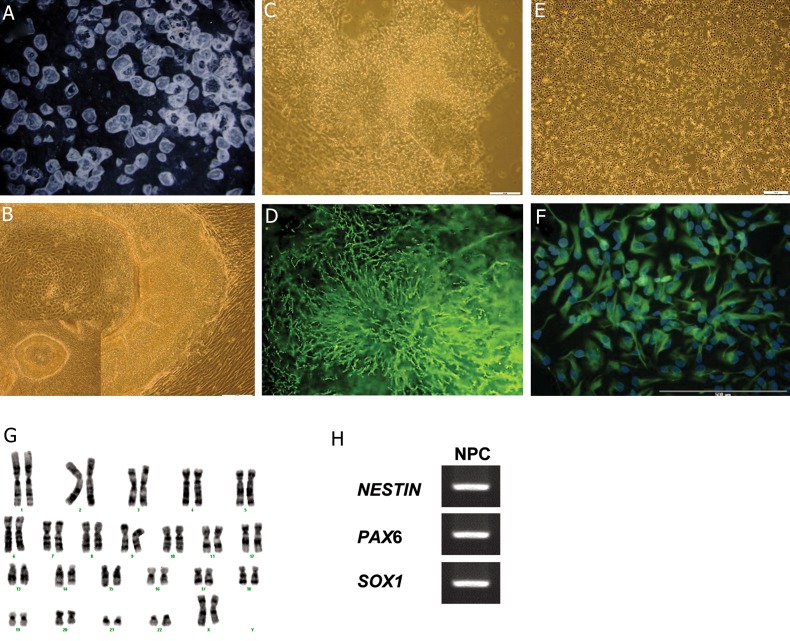
Induction of human embryonic stem cells (hESCs) into neural cells by co-culture with stromal stem cells from human exfoliated deciduous teeth (SHED). A. Stereo photomicrographs of hESC colonies with central crater-like structures, B. Numerous neural tube-like structures located in the margin of the colonies on day 14, C. Neural progenitor cells (NPCs) with rosette-like structures before passaging, D. were ZO1 (epithelial marker) positive , E. Adherent culture of NPCs , F. was NESTIN positive, G. NPCs showed normal karyotype and H. expressed NESTIN, SOX1 and PAX6.

Within the first days of seeding hESCs on SHEDs, we observed morphological changes to the neuroectoderm created by the formation of crater-like structures with numerous rosettes in the margins of the hESC colonies. These structures led to the formation of neural tubes by day 14 (Fig.1A, B). Rosette-like structures (Fig.1C) were ZO1 immunopositive (Fig.1D). After additional passages these NPCs grew as a monolayer (Fig.1E) and expressed NESTIN (Fig.1F). These neural cells had a normal karyotype (Fig.1G) and expressed the NPC markers, *SOX1, PAX6* and *NESTIN* (Fig.1H). The expressions of general mesodermal markers, *GATA4* and *BRACHYURY*, and endodermal markers, *FOXA2* and *SOX17*, were not detected in the differentiated cells (data not shown).

**Fig.2:**
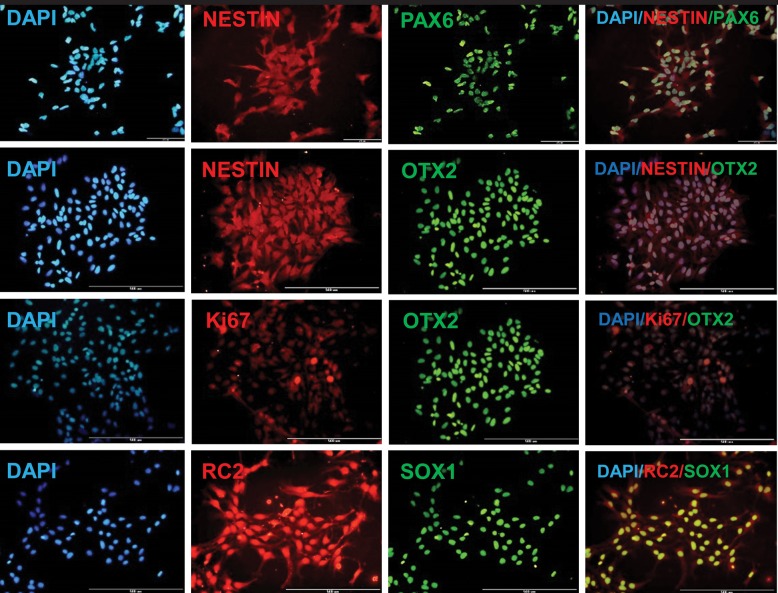
Immunofluorescence staining of human embryonic stem cell derived neural precursor cells (hESC-NPCs). Rostral identity and proliferation potency showed by immuno co-staining for NESTIN/OTX2, NESTIN/PAX6 and OTX2/Ki67. Neuroepithelial and radial glia characteristic demonstrated by RC2/SOX1 immuno co-staining.

The NPCs co-expressed anterior neural markers, NESTIN/PAX6 and NESTIN/OTX2. Their proliferation potency was confirmed by co-expression of OTX2/Ki67. The neuroepithelial identity of these cells was verified by RC2 expression, as a marker of neuroepithelium and radial glia, and SOX1, a transcription factor expressed by definitive neuroepithelial cells during neural tube closure (Fig.2). 

### hESCNPCs showed neural crest progenitor cell characterizations

**Fig.3:**
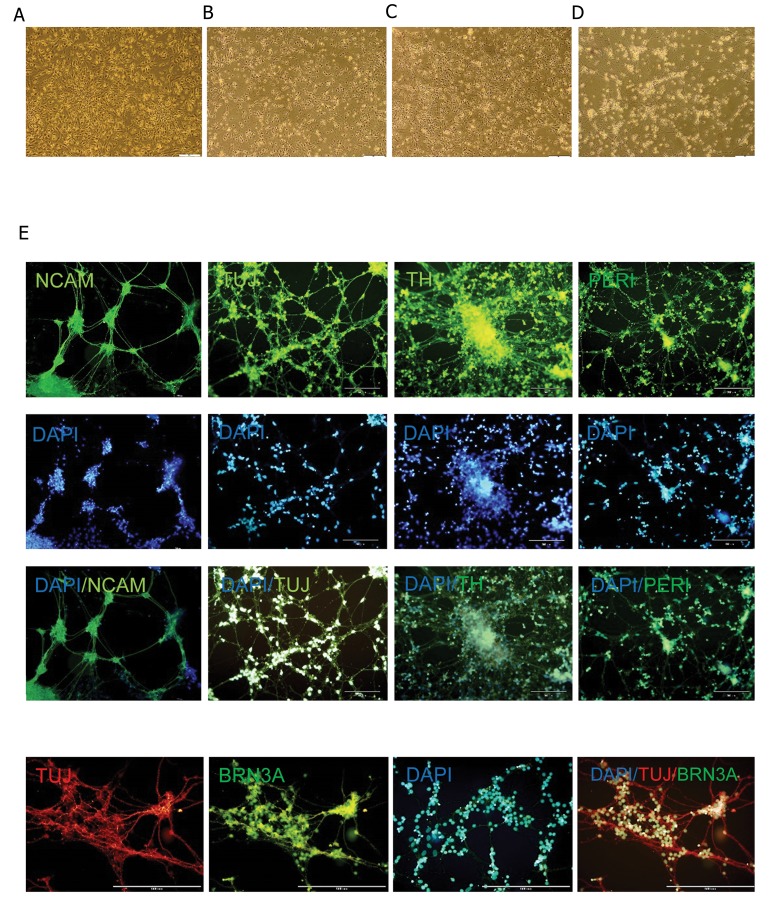
Phase contrast (A-D) and immunofluorescence staining (E) of differentiated human embryonic stem cell derived neural progenitor cells (hESC-NPCs). Visible network structures appeared following a long culture period of neural cells that had bipolar morphology and distinct soma. The differentiated cells were positive for TUJ, NCAM mature neural markers and TH, PERIPHERIN and BRN3A as markers of peripheral neurons.

Neural induction in the absence of growth factor supplementation resulted in neural cells that had bipolar morphology and distinct soma. After additional culture the soma were aggregated whereas the neural process elongated and formed clearly visible network structures. These cells were positive for mature neural markers, NCAM and TUJ, as well as TH, PERIPHERIN and BRN3A, as peripheral neurons markers (Fig.3). 

**Fig.4:**
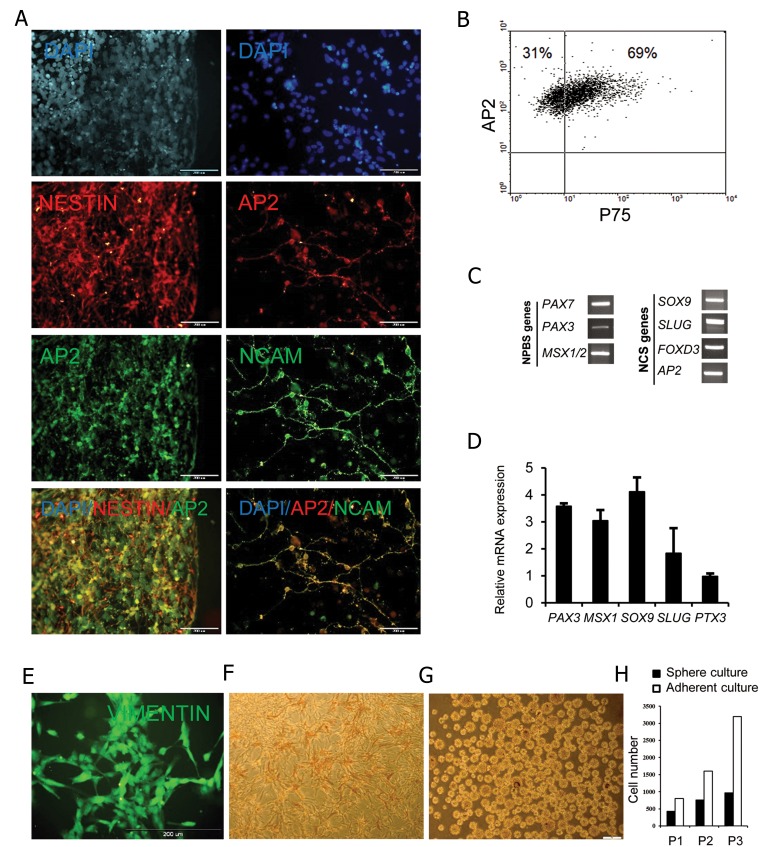
Characterization of neural crest identity in human embryonic stem cell derived neural progenitor cells (hESC-NPCs). A. Immunofluorescence staining showed double expression of NESTIN/AP2 and AP2/NCAM as markers of neural crest cells, B. Flow cytometry analysis demonstrated high expression of AP2/P75, C. RT-PCR and D. real-time analyses. Neural plate border specifier (NPBS) and neural crest specifier (NCS) genes, E. Immunofluorescence staining for VIMENTIN, F. Alizarin red staining showed osteogenic differentiation on induced cells, G. Proliferation of the cells as neurosphere on ultra-low attachment plates and H. Expansion of the cells in adherent and neurosphere cultures.

The results showed that these cells were positive for markers of neural crest cells AP2/NCAM (Fig.4A). According to flow cytometry analysis, approximately 70% of these cells co-expressed AP2 and P75, another marker that defined neural crest cells (Fig.4B). Assessment of expression of neural plate border specifier (NPBS) genes, *PAX3/7* and *MSX1/2*, and neural crest specifier (NCS) genes *SOX9, SNAIL2/ SLUG, FOXD3*, and *AP2* revealed that these cells were positive for NPBS and NCS markers (Fig.4C) and negative for later-expressed neural crest effector genes (*c-KIT* and *c-RET*, data not shown). These neural crest progenitor cells (NCPCs) expressed *FOXD3* and *SNAIL2/SLUG*, characteristic of neural crest cells (Fig.4C). The expression of some of these neural crest markers were further verified by real-time PCR (Fig.4D). 

These cells were positive for VIMENTIN (Fig.4E) (11) and were alizarin red positive when cultured in osteogenic media (Fig.4F). Furthermore, they readily formed neurospheres upon culture in neural stem cell medium in non-adhesive dishes (Fig.4G). Proliferation rate in adherent cultures was more than the proliferation rate in suspension cultures (Fig.4H). 

### Discussion

Neural induction can be induced through several methods, including the co-culture of hESCs with cells that have SDIA activity. Considering that SHED cells have stromal origin and express stromal cell markers, in this study we have evaluated their potential as an SDIA source. To achieve this, hESCs were co-cultured with SHED cells as reported for other cell lines with SDIA (3-7). During the first days of this co-culture period we observed morphological changes on hESC colonies. On day 14 there were large numbers of typical neural tube-like structures observed at the margins of these colonies. Interestingly, in addition to the different appearance of the hESC colonies (crater appearance), we also observed rosettes and neural tube-like structures in the periphery of the hESCs colonies. In other studies, researchers observed these structures in the center of the hESC colonies when SDAI lines such as PA6 and MS5 were used as a feeder layer for the co-cultures (3, 4, 12). Possibly this observation could be related to the different source of SDIA.

NPCs derived from these neural tube-like structures expressed early neural markers (*PAX6, SOX1*) and *NESTIN*. For molecular analysis, co-cultures were digested with TrypLE™ Express and the resultant cell suspension was cultured in NPC specific culture medium. Of note, it has been established that SHED-like cells cannot proliferate in serum-free medium (13).

Whether these cells have bone morphogenic protein (BMP) antagonistic behavior or they induce neurogenesis activity through a different mechanism remains to be answered. In this regard Kawasaki et al. (8) have used an antibody for the BMP receptor to show that BMP antagonism is unlikely to explain SDIA. Recently, Vazin et al. (14) used microarray techniques and have reported that SDIA is exerted by secretion of novel combined factors that consist of stromal-derived factor, pleiotropin, insulin-like growth factor and ephrin B1.

Gene expression analysis of these cells showed that these cells expressed anterior neural and neural crest markers. In addition, these NCPCs expressed NPBS and NCS genes but not later-expressed neural crest effector genes such as *c-KIT* and *c-RET* (15). The latter two markers are believed to be expressed by migrating neural crest cells induced by the presence of BMP antagonists (16). It has been reported that BMP antagonists are not produced by SDIA cell lines (8, 17). In the current study, the cells were not treated with a BMP antagonist required for migration of neural crest cells. Therefore, the lack of expression of these two markers was not unexpected. Expression of NCPC marker by NPCs may be related to intrinsic properties of SHED that express neural crest markers *PAX3, MSX1/2, SNAIL/SLUG* and *AP2*. Expression of VIMENTIN as a neural stem cell marker and alizarin red staining have shown that these cells have cranial specification.

These cranial NCPC were easily passaged compared to other NCPCs. Cranial NCPCs had more proliferation rate and self-renewal activity than trunk neural crest precursor cells (18). In addition, when these cells were cultured in suspension they formed uniform spheres of a similar size but their proliferation capacity decreased with further passages as previously reported (4, 5). Pomp et al. (4) also observed similar results and suggested that this phenomenon was likely related to gliogenesis in these neurospheres.

TH positive cells have generally been obtained when NPCs are treated with SHH, FGF8, or stromal conditioned medium (17, 18). Of interest, nearly all NPCs obtained in this study that underwent differentiation in neural induction media were positive for NCAM, TUJ and TH without the need for inductive factors such as SHH, FGF8 or stromal conditioned medium. This suggested that these cells were already specified to form TH positive cells during co-culture with SHED cells. In addition, our cells were positive for PERIPHERIN and BRN3A markers of peripheral nerves derived from NCCs. Expression of this combination of markers was thought to be an almost exclusive characteristic of peripheral sensory neurons (PSNs) (19).

### Conclusion

Our results, for the first time, provide evidence that SHED cells have stromal inducing activity and NPCs with neural crest specification. A high presentation of TH activity can be derived in a relatively short period of time from co-culturing hESCs with these cells. Apparently the ability to induce NPCs is common to all SDIA lines; however, more importantly, SHEDs induce neural crest markers which have not been previously reported in other SDIA lines. This difference may account for neural crest specification of NPCs.
