# Stem Cells from Human Exfoliated Deciduous Tooth Exhibit Stromal-Derived Inducing Activity and Lead to Generation of Neural Crest Cells from Human Embryonic Stem Cells 

**DOI:** 10.22074/cellj.2021.7931

**Published:** 2021-03-01

**Authors:** Khadijeh Karbalaie, Somayyeh Tanhaei, Farzaneh Rabiei, Abbas Kiani-Esfahani, Najmeh Sadat Masoudi, Mohammad Hossein Nasr-Esfahani, Hossein Baharvand

**Affiliations:** 1. Department of Cellular Biotechnology at Cell Science Research Center, Royan Institute for Biotechnology, ACECR, Isfahan, Iran; 2. Department of Molecular Biotechnology at Cell Science Research Center, Royan Institute for Biotechnology, ACECR, Isfahan, Iran; 3. Department of Genetics at Reproductive Biomedicine Research Center, Royan Institute for Reproductive Biomedicine, ACECR, Tehran, Iran; 4. Department of Stem Cells and Developmental Biology at Cell Science Research Center, Royan Institute for Stem Cell Biology and Technology, ACECR, Tehran, Iran; 5. Department of Developmental Biology, University of Science and Culture, ACECR, Tehran, Iran

In this article which was published in Cell J, Vol 17, No 1, Spring 2015, on pages 37-48, we found that Figure 1H, Figure 2 (OTX2, row 3), and Figure 3 (row 4) had been published incorrectly. The following figures are corrected.

The authors would like to apologies for any inconvenience caused.

**Fig.1:**
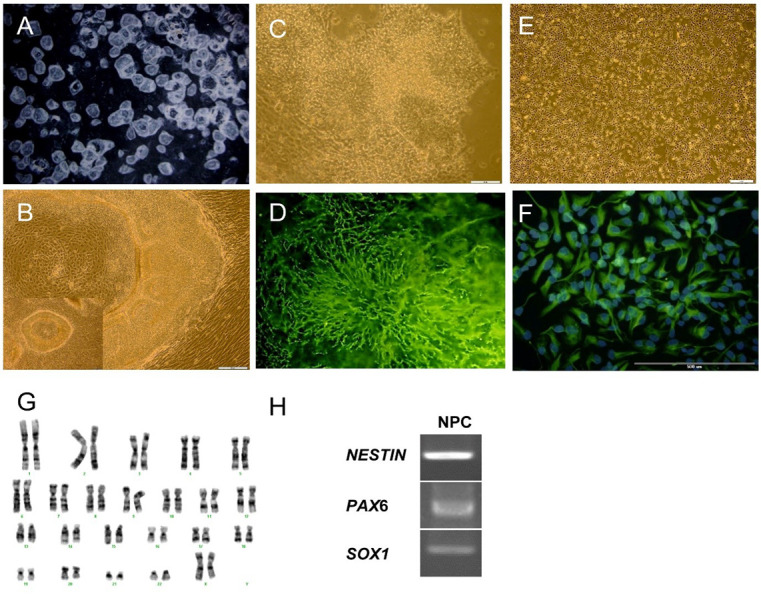
Induction of human embryonic stem cells (hESCs) into neural cells by co-culture with stromal stem cells from human exfoliated deciduous teeth (SHED). **A.** Stereo photomicrographs of hESC colonies with central crater-like structures, **B.** Numerous neural tube-like structures located in the margin of the colonies on day 14, **C.** Neural progenitor cells (NPCs) with rosette-like structures before passaging, **D. **were ZO1 (epithelial marker) positive , **E.** Adherent culture of NPCs , **F. **was NESTIN positive, **G. **NPCs showed normal karyotype and **H.** expressed *NESTIN, SOX1 and PAX6*.

**Fig.2:**
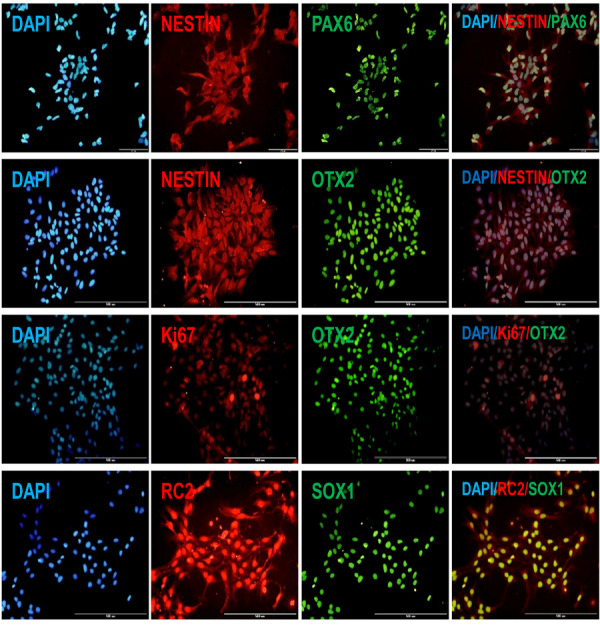
Immunofluorescence staining of human embryonic stem cell derived neural precursor cells (hESC-NPCs). Rostral identity and proliferation potency showed by immuno co-staining for NESTIN/OTX2, NESTIN/PAX6 and OTX2/Ki67. Neuroepithelial and radial glia characteristic demonstrated by RC2/SOX1 immuno co-staining.

**Fig.3:**
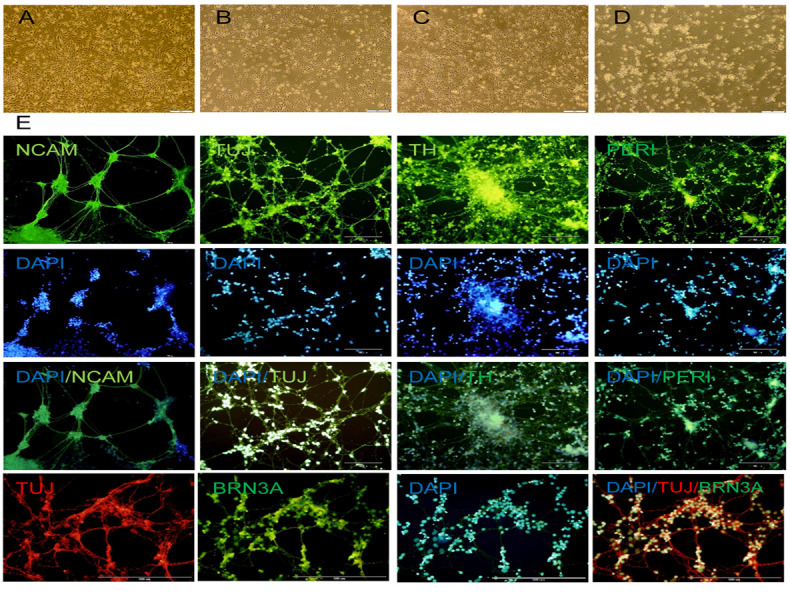
Phase contrast **(A-D)** and immunofluorescence staining **(E)** of differentiated human embryonic stem cell derived neural progenitor cells (hESC-NPCs). Visible network structures appeared following a long culture period of neural cells that had bipolar morphology and distinct soma. The differentiated cells were positive for TUJ, NCAM mature neural markers and TH, PERIPHERIN and BRN3A as markers of peripheral neurons.

